# 559 Nursing Management of Multiple Concurrent Pediatric Patients with Cultured Epidermal Autografts

**DOI:** 10.1093/jbcr/irac012.187

**Published:** 2022-03-23

**Authors:** Christine M Grauer, Miranda L Yelvington

**Affiliations:** Arkansas Children's Hospital, Little Rock, Arkansas; Arkansas Children’s Hospital, Little Rock, Arkansas

## Abstract

**Introduction:**

For patients with large areas of full-thickness burns and limited autologous donor sites, cultured epidermal autografts (CEAs) can provide life-saving coverage. Nursing care of a patient with CEAs can be challenging due to the fragility of these grafts and compounded by the critical status of the patient. Care of a single patient with CEAs can prove challenging, but in cases in which multiple patients receive CEAs concurrently, these challenges are amplified.

**Methods:**

Three pediatric patients with large burn injuries were admitted on a single day to a large academic hospital with a Burn Center (Table 1).

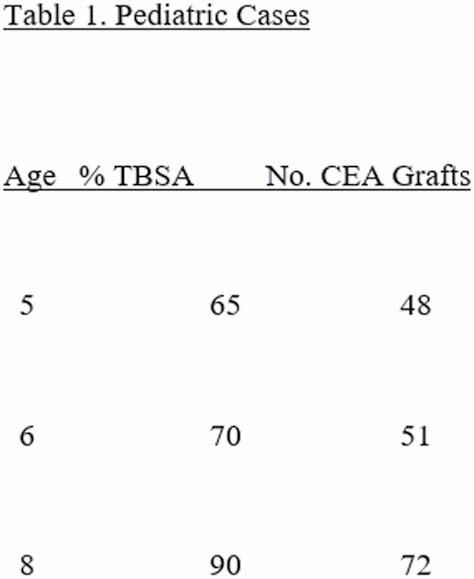

Nursing care of these children with concurrent CEA placement required an all-hands-on-deck approach and collaboration between the Burn Center (BC) and the Pediatric Intensive Care Unit (PICU) with regards to staffing, care, and positioning. Consistent primary RNs from both the BC and PICU staffed patients and volunteered to work additional shifts during a period of high census. To meet patient needs, the multi-disciplinary care team developed a schedule for CEA exposure, wound care, and therapy interventions.

**Results:**

To meet the needs of these patients under the current staffing pattern, a team schedule was developed (Table 2).

Table 2. Care Schedule

0300-0400 Night shift staff removed anterior and extremity dressings with patients in supine.

0800-0900 Therapy staff arrived early during the day shift to perform therapy interventions with anterior CEAs exposed. Multi-disciplinary teams applied anterior and extremity dressings, positioned patients prone, and removed dressings from posterior CEA sites.

1700-1800 After approximately six to eight hours of CEA exposure, teams reapplied dressings and positioned patients supine.

**Conclusions:**

Staff followed this schedule for these patients until their CEAs were determined to be integrated. Planning and collaboration among all members of the treatment team are integral to the successful care of pediatric patients with CEAs. A schedule for wound care, turning, and CEA exposure can improve staff communication, ease hand-offs, and ensure optimal quality of care for multiple patients with these large burn injuries.

